# Complex tourism and season interactions contribute to disparate physiologies in an endangered rock iguana

**DOI:** 10.1093/conphys/coac001

**Published:** 2022-02-05

**Authors:** Susannah S French, Alison C Webb, Travis E Wilcoxen, John B Iverson, Dale F DeNardo, Erin L Lewis, Charles R Knapp

**Affiliations:** 1 Department of Biology, Utah State University, Logan, UT 84322, USA; 2 Ecology Center, Utah State University, Logan, UT 84322, USA; 3 Biology Department, Millikin University, Decatur, IL 62522, USA; 4 Department of Biology, Earlham College, Richmond, IN 47374, USA; 5 School of Life Sciences, Arizona State University, Tempe, AZ 85281, USA; 6 Daniel P. Haerther Center for Conservation and Research, John G. Shedd Aquarium, Chicago, IL 60605, USA

**Keywords:** reproduction, oxidative stress, immunity, glucose, Cyclura, corticosterone

## Abstract

To promote survival and fitness, organisms use a suite of physiological systems to respond to both predictable and unpredictable changes in the environment. These physiological responses are also influenced by changes in life history state. The continued activation of physiological systems stemming from persistent environmental perturbations enable animals to cope with these challenges but may over time lead to significant effects on the health of wildlife. In the present study, we tested how varying environmental perturbations driven by tourism and associated supplemental feeding affects the energetics, corticosterone and immunity of six discrete populations of the northern Bahamian rock iguana (*Cyclura cychlura inornata* and *Cyclura cychlura figginsi*). We studied populations within and outside the reproductive season and quantified tourist numbers during sample collection. Specifically, we measured clutch size, body condition, plasma energy metabolites, reactive oxygen species, baseline corticosterone concentrations and immune function of male and female iguanas from each population to address whether (i) disparate physiologies are emerging across a gradient of tourism and feeding, (ii) both subspecies respond similarly and (iii) responses vary with season/reproductive condition. We found significant effects of tourism level, season and their interaction on the physiology of both *C. c. inornata* and *C. c. figginsi*, supporting the idea that tourism is leading to the divergence of phenotypes. Specifically, we found elevated plasma energy metabolites, oxidative stress and a measure of innate immunity (bactericidal ability), but reduced corticosterone concentrations with increasing tourism in both subspecies of rock iguanas. These physiological metrics differ according to the level of tourism in both subspecies and persist across seasons despite variation with natural seasonal and reproductive changes. These findings suggest that anthropogenic disturbance results in disparate physiologies in northern Bahamian rock iguanas.

## Teaser

We studied the impact of tourism and supplemental feeding on populations of endangered iguanas at different times of the year. Although the physiology of animals varied across seasons, tourist activities significantly impacted all physiological measures regardless of season. Tourism may therefore lead to lasting effects on these endangered iguanas.

## Introduction

An organism’s physiology is inextricably linked to its environment, whereby an animal’s physiology enables it to cope with a variable landscape of perturbations (Wingfield and Kitaysky, 2002; Wikelski and Cooke, 2006). There is a suite of mechanisms that organisms use to respond to both predictable (i.e. seasonal changes) and unpredictable (i.e. challenges that trigger a stress response) changes in their environment. Of utmost significance are endocrine mediators that alter energetics and metabolism, including sex steroids, to initiate and regulate the reproductive process in all vertebrates (Tilbrook *et al.,* 2002; Norris and Carr, 2020) and glucocorticoids that mobilize energy in response to environmental stressors (Romero and Butler, 2007; Saplosky, 1992; Sapolsky *et al.,* 2002). In addition, key downstream metrics that reflect the consequences of primary physiological responses also pose simultaneous health costs, including oxidative stress and immune function (Grossman, 1984; Costantini, 2008; Dhabhar, 2009a; Dhabhar, 2009b; Costantini *et al.,* 2011).

The continued activation of physiological systems that enable animals to cope with environmental challenges may lead to disparate physiological phenotypes both in terms of circulating hormone levels and their downstream effects. As a result, differing phenotypes or eventually genetically distinct ecotypes may emerge whereby we see consistent differences in morphology, behaviour and/or physiology across variable environmental landscapes (Angilletta, 2001; Bronikowski and Vleck, 2010). A classic example of this is the garter snake system in the Sierra Nevada Mountains, CA, USA, in which consistently different life histories persist across elevational differences (Bronikowski and Vleck, 2010). This natural elevational variation elicits different thermal environments as well as resources for populations of garter snakes across the montane landscape. The resulting life history divergences lead to population differences in body size, metabolism, reproductive output, lifespan (Bronikowski and Vleck, 2010), reproductive investment and senescence (Sparkman *et al.,* 2007), corticosterone release in response to stress (Palacios *et al.,* 2012) and immune investment (Sparkman and Palacios, 2009; Palacios *et al.,* 2011). While studies have demonstrated the emergence of ecotypes in response to natural variation, less work has focused on human-caused environmental changes that could also result in the emergence of ecotypes. This is especially relevant because anthropogenic pressures increasingly present new changes and challenges for wildlife.

Ecotourism is a growing industry that leads to increased human-wildlife interactions and subsequent consequences. Ecotourism is touted for positively affecting human attitudes towards the conservation of nature and for supporting local economies (Hunt *et al.,* 2015; Boley and Green, 2016). However, human–animal interactions associated with ecotourism, including handling, observing, photographing and food provisioning, may have unintended consequences for the wildlife involved (Flather and Cordell, 1995; Stewart *et al.,* 2016). Studies have been limited, but profound effects on animal behaviour, physiology and even survival have been documented (Iverson *et al.*, 2004a; Hines, 2011; Knapp *et al.,* 2013, Smith and Iverson, 2015). For example, a long-term demography study in Bahamian rock Iguanas showed that individual growth rates increased and population density increased over time on tourist-visited cays (Iverson *et al.,* 2006; Smith and Iverson, 2016). These types of differences have been found in other systems, resulting in a variety of impacts, including altered animal behaviour (Walker *et al.,* 2005; Walker *et al.,* 2006; Villanueva *et al.,* 2012), modification of the hypothalamic pituitary–adrenal axis release of hormones—such as corticosterone, in response to challenges that elicit stress (Romero and Wikelski, 2002; Ellenberg *et al.,* 2007; French *et al.,* 2017)—changes in body condition and physiology (Knapp *et al.,* 2013; Smith and Iverson, 2015) and changes in survival (Müllner *et al.,* 2004; Iverson *et al.,* 2006). Thus, it is possible that chronic interpopulation differences in anthropogenic influence on the environment may be akin to natural variations that lead to the divergence of phenotypes, both behavioural and physiological.

Rock iguanas (*Cyclura cychlura*) in The Bahamas offer an exemplary system to address the effects of anthropogenic pressures on physiology by testing whether long-standing anthropogenic influences can induce physiological changes in free-ranging animals. In this model system, tourist visitation and supplemental feeding of iguanas is consistently different on adjacent cays that are otherwise similar. Animals at high tourist sites are visited and fed by hundreds of people daily, whereas moderate tourist sites only received approximately 18 visitors a day on average, and iguanas from unvisited sites are not fed by humans at all. Tourists primarily feed fruit on skewers, with grapes being to most frequent food item. This provides an ideal natural experiment to study the impact of disturbance and altered diet on physiology and reproduction, especially since altered diet and resource availability have been shown to have profound effects on other wildlife (Orams, 2002; Barnett *et al.,* 2016; Stewart *et al.,* 2016). Moreover, there is preliminary evidence that tourism may be inducing the emergence of disparate behavioural and physiological phenotypes (Iverson *et al.,* 2006; Hines, 2011; Knapp *et al.,* 2013). Previous studies with these lizards have demonstrated that tourism significantly alters feeding behaviour, desensitizes rock iguanas to humans (Hines, 2011), changes blood chemistry (including glucose; Knapp *et al.,* 2013), increases growth and body condition (Smith and Iverson, 2016) and influences population density and individual survival (Iverson *et al.,* 2006; Smith and Iverson, 2016). These insights provide an ideal foundation for expanding the work to include ecoimmunological and oxidative health indicators.

Immunological markers can provide important insight regarding the health of organisms (Demas *et al.,* 2011; French and Neuman-Lee, 2012; French *et al.,* 2017). Investigating how animals can functionally respond to a relevant pathogen (without infecting the animal) provides information regarding the actual health consequences of perturbations. For example, we utilize a bacterial killing assay that provides a functional assessment of immunity involving actions of haemolytic complement, antimicrobial peptides, opsonizing proteins and natural antibodies (Demas *et al.,* 2011; French and Neuman-Lee, 2012; French *et al.,* 2017). This is a step beyond simply looking at whether or not there is an effect and that may lead to an understanding of the implications for animal health of perturbations. Likewise, corticosterone (the primary glucocorticoid in reptiles) and oxidative stress can increase during times of chronic stress and have been linked to reduced survival if chronically elevated (Romero and Wikelski, 2001; Costantini *et al.,* 2011; Lucas and French, 2012; Costantini and Dell'Omo, 2015 ). However, sufficient antioxidants may help abate the effects of oxidative stress and so measuring relative amounts of reactive oxygen metabolites to antioxidants will provide a more comprehensive look at oxidative status (Vassalle, 2008). In addition, energy status can help inform condition and how well animals may respond to perturbations. Circulating energy metabolites may then provide additional information about energetics that is not obvious via body condition measurements alone (Knotek *et al.,* 2003; McGuire *et al.,* 2009; Fokidis *et al.,* 2012; Neuman-Lee *et al.,* 2015). Finally, in wild populations fed by humans, energy metabolites in the blood may also inform dietary health of animals (Knapp *et al.,* 2013).

The physiological state of an animal is also highly dependent on its life history and natural environmental changes that occur on predictable scales. Perhaps most notable are changes that occur seasonally to facilitate reproduction—the ultimate fitness metric. For example, circulating hormone concentrations, mobilization of energy stores and even immune function all vary with reproductive state (Bilbo and Nelson, 2003; French and Moore, 2008; Webb *et al.,* 2019; Zimmerman *et al.,* 2010). Significantly, the same physiological responses that mediate predictable reproductive events are also key to responding to unpredictable challenges. Therefore, naturally occurring changes in life history and subsequent physiological states can significantly impact how an animal responds to environmental perturbations, including the tourist-induced effects we investigated.

We tested how varying tourism levels and associated supplemental feeding affect the energetics, stress and immunity of six discrete populations of Bahamian rock iguanas within and outside the reproductive season. Our study sites represent all known reproducing populations of the northern subspecies of northern Bahamian rock iguanas (*C.* c. *inornata*), except the introduced population on Alligator Cay (Knapp, 2001), and the majority of known populations of the southern subspecies (*C.* c. *figginsi*), making the results valuable to enhance our fundamental understanding of iguana health, its implications for affected wildlife and to inform conservation management. We assessed current visitation rates and specifically measured clutch size, body condition, plasma energy metabolites, reactive oxygen species, baseline corticosterone concentrations and innate immunity of male and female iguanas from each population to address the following questions: (i) Are disparate physiologies emerging across a gradient of tourism and feeding? (ii) Are both subspecies responding similarly? (iii) How do the responses vary/interact with season/reproductive condition? We predicted that physiological measures across populations would correspond to the degree of tourism and supplemental feeding and differences would be replicated in the northern and southern subspecies. We also predicted that these responses would shift as a result of reproductive activity, leading to interactions between season and tourism exposure.

## Materials and methodology

### Study design

Northern Bahamian rock iguanas, *C. cychlura,* are distributed in isolated populations on Andros Island and a small number of cays of the Exuma island chain on the Great Bahama Bank (Malone *et al.,* 2003; Hines, 2017). The Exuma populations are separated into two geographically disjunct (by 80 km) subspecies, *C. c. inornata* and *C. c. figginsi*, and are listed as Critically Endangered according to IUCN Red List of Threatened Species™ criteria (Knapp, 2004; Iverson *et al.,* 2019; Colosimo *et al.,* 2021). For each subspecies, we studied a three-cay cluster of adjacent cays, whereby each cay in a cluster has similar habitats yet experiences considerable differences in tourism and supplemental feeding. For each cluster there is a cay that is highly visited, a cay with moderate visitation and an unvisited cay (not visited by anyone due to difficulty of access). Disturbance is easily quantifiable via the number of boats and people that visit these uninhabited cays (Knapp, 2004; Iverson *et al.,* 2006). Genetic data from both subspecies suggest that there has been little migration between populations following fragmentation by rising sea levels in the Pleistocene (Malone *et al.,* 2003; Aplasca *et al.,* 2016).

*Cyclura cychlura inornata* begins mating in early to mid-May and migrates to nesting sites in mid to late June where females dig nests and defend them for at least 3–4 weeks after oviposition (Iverson *et al.*, 2004b). On average, one in three adult females nest each year producing a clutch of 1–10 eggs, with frequency of reproduction and clutch size increasing with female size (Iverson *et al.,* 2004b). The southernmost populations of *C. c. figginsi* included in this study begin the breeding season 1–2 weeks earlier (Iverson *et al.*, unpublished).

In 2016, we haphazardly sampled adult rock iguanas (i.e. SVL over 24 cm) via capture by hand, dipnet or snare connected to a retractable pole. We sampled within the reproductive season from 18–24 May (male, 70; female, 63) to 20–26 June (male, 94; female, 78) and outside the reproductive season from 3 to 11 September (male, 93; female, 86) from a high-, moderate- and no-tourism site located in the northern Exuma Islands (*C. c. inornata*) and a high-, moderate- and no-tourism site located in the southern Exuma Islands (*C. c. figginsi*) in The Commonwealth of The Bahamas. All pursuits, captures and times to blood collection were recorded for each animal. The majority of capture and bleed times were under 3 minutes and so we saw no effect of capture time on physiological parameters measured (Tylan *et al.,* 2020). Due to travel logistics the southern sites were only sampled in June and September. We sexed animals using a cloacal probe, unless a hemipenis was confirmed visually.

We ranked sites as either ‘high tourism’ (average, 131 tourists/day), ‘moderate tourism’ (average, 18 tourists/day) or ‘no tourism’ (no tourists) based initially on our estimation of the intensity of human presence on each of the cays and quantified during our visits in 2019 as the number of visitors per day averaged over the sampling time frame. These ranking were based on both volume of tourism and regularity of visits, whereby high-tourism sites experience consistent daily visits from multiple tour companies with large numbers of people, weather permitting. Moderate-tourism sites experience less organized tourist visits and are visited intermittently by significantly fewer numbers of people from private yachts or smaller, personalized tour groups. To our knowledge, no-tourism sites experience no visitors due to the extreme difficulty of cay access (no landing beaches). This was substantiated by the skittish behaviour of the iguanas on these cays. These rankings are further supported by over 25 years of annual personal observations at each site.

### Physiological metrics

#### Sample collection

We collected blood samples following the procedures described in Webb *et al.* (2019). Our capture and collection methods allowed for true baseline sampling of individual physiology, independent of handling and capture stress (Delehanty and Boonstra, 2012). We collected blood samples from the caudal vein between 0800 and 1300 hours with an average time to bleed of 110 ± 61 seconds (SD) and a max time of ~270 seconds. There was no correlation between any physiological measure and time to acquire the blood sample (*r*^2^ < 0.06). We calculated body condition as the residual from a snout–vent length–mass ordinary least squares regression (Schulte-Hostedde *et al.,* 2005).

#### Plasma energy metabolites

We measured two lipid metabolites (triglycerides and free glycerol) via sequential enzymatic colour endpoint assays (F6428, T2449 and G7793; Sigma-Aldrich, MO, USA) using the manufacturer’s instructions and a dilution protocol (Guglielmo *et al.,* 2002) to enable use of a 96-well microplate with a 5-minute incubation at 37°C. Following the protocol described in Webb *et al.* (2019), we ran 5 μl samples from each individual in duplicate to sequentially measure free glycerol and triglycerides. The mean coefficient of variation (CV) across assay plates was 4.88% and inter-assay CV was 4.08%.

To reduce the number of factors in the analysis and reduce error by including variables that are highly correlated in the models, we used principle component analysis to create a triglyceride index. Total triglycerides, true triglycerides and free glycerol were included in this factor reduction, resulting in a principal component 1 with an eigenvalue of 87.7%. This index was used as one of the dependent variables in the analysis.

#### A measure of innate immunity: bacterial killing assay

We used a bacterial killing assay involving the actions of opsonizing proteins, antimicrobial peptides and natural antibodies to assess the ability of blood plasma to eliminate a pathogenic bacterium, *Escherichia coli*. This technique provides a functionally relevant measure of host immune function (Tieleman *et al.,* 2005; French and Neuman-Lee, 2012). Following the protocol described in French and Neuman-Lee (2012), we diluted plasma at 1:12 with 0.9% phosphate-buffered saline (PBS) and combined with CO_2_-Independent Medium (Gibco, Grand Island, NY, USA) plus 4 nM L-glutamine (Sigma-Aldrich), 10^5^ CPU (colony producing unit) *E. coli* (EPowerTM Microorganisms #0483E7, ATCC 8739, MicroBioLogics, St. Cloud, MN, USA) and tryptic soy broth on a 96-well microplate. We ran all samples in duplicate. We calculated background absorbance using a BioRad xMark microplate reader. After a 12-hour incubation at 37°C, we read absorbance following bacterial growth. We calculated bactericidal ability by subtracting background absorbance to account for any difference in plasma absorbance values, we averaged replicates for each control and sample and then divided the absorbance for each sample by the absorbance for the positive controls (containing only medium and bacterial solution) [e.g. (1-(mean absorbance of sample/mean absorbance of positive controls))*100]. This provides the percent bacteria killed relative to the positive controls. We ran negative controls (containing only medium) to ensure that there was no background contamination. The mean CV across all plates was 4.37% and the CV was <5.98% on all plates.

#### Radioimmunoassays

We measured baseline circulating concentrations of corticosterone (both sexes; Ab: MP Biomedicals 07-120 016), estradiol (females only; Ab: Biogenesis 7010-2650), progesterone (females only; Ab: Fitzgerald 20R-PR053W) and testosterone (males only; Ab: Fitzgerald 20R-TR018w) using a radioimmune assay. We performed assays following a protocol previously described by French *et al*. (2010) (Moore *et al.,* 1991). We extracted samples with isooctane and ethyl acetate solution, dried down and resuspended in PBS. We assayed all samples in duplicate and used the mean for analysis. We used individual recoveries for each sample to adjust final concentration values following extraction. For corticosterone in both sexes, the mean CV was 10.97%. In females, the mean CV for progesterone was 17% and for estradiol it was 10.05%. In males, the mean testosterone CV was 11.9%.

#### Reactive oxygen metabolites and antioxidant capacity

We assessed oxidative physiology by measuring the derivatives of reactive oxygen metabolite species (d-ROMs) and the effectiveness of antioxidant defences. Following methods described in Webb *et al.* (2019), we quantified circulating d-ROMs and antioxidant capacity using commercially available assay kits (MC435 and MC002; Diacron International, Italy). We diluted plasma in the provided acidic buffered solution (5 μl: 100 μl) for the d-ROMs assay and in distilled water (2 μl: 100 μl) for the antioxidant assay. We ran all samples in duplicate. The mean d-ROMs CV across all assays was 3.66%, and all CVs were <4.49%. The mean antioxidants CV was 7.08%, and all CVs were <11.98%. We calculated an oxidative index by subtracting a standardized antioxidant value (antioxidant value − mean antioxidants)/standard deviation) from a standardized d-ROMs value, representing the relative contribution of d-ROMs and antioxidant capacity. Higher values are associated with elevated levels of oxidative stress (Vassalle *et al.,* 2008).

#### Reproduction

Reproduction requires significant energy and nutritional resources, resulting in substantial changes to an animal’s overall physiology and behaviour. Thus, in trying to understand the impact of human activities such as tourism, reproductive status can have profound consequences. Oviparous animals such as iguanas deposit all of their energy into easily quantifiable units—eggs. Using high-resolution ultrasonography (Sonosite iViz, Bothell, WA, USA) we quantified total clutch volume (both number and size of follicles and eggs).

## Statistical analysis

Progesterone, estradiol, testosterone and corticosterone values were not normally distributed; therefore, these values were log_10_-transformed prior to inclusion in the statistical models. We used multivariate analysis of covariance (MANCOVA) to assess variation in physiology among iguanas, with separate models for each subspecies and separate models for each sex within each subspecies. Subspecies were included in separate models because of the known variation in the timing of breeding and unequal replication in sampling between subspecies. Further, the same suite of physiological products was not assessed for female and male iguanas, warranting different statistical models for each. There was moderate correlation among each of the dependent variables used in the models (absolute values of Pearson’s r ranged from 0.24 to 0.71). We completed tests of multicollinearity and found the greatest variance inflation factor to be 2.602 (body condition index and bacterial killing ability).

For female iguanas, we included the continuous dependent variables of progesterone (log-transformed), estradiol (log-transformed), triglyceride index, bacterial killing ability (log-transformed), corticosterone (log-transformed) and oxidative index in the MANCOVA models. We included sample month (June and September for *C. c. figginsi*; May, June and September for *C. c. inornata*), tourism (none, moderate, high) and the interaction term for sample month*tourism as independent group variables. We included body condition as a covariate in all models. For models in which the sample month*tourism interaction was significant, we completed separate MANCOVA’s within each of the months. We also added ovarian follicle status (yes or no) as an independent variable for the month in which female iguanas were in reproductive condition.

For male iguanas, we included testosterone (log-transformed), triglyceride index, bacterial killing ability (log-transformed), corticosterone (log-transformed), and oxidative index in the MANCOVA models. These included sample month, tourism and the sample month*tourism interaction (as described above for females of each subspecies) as independent variables. We included body condition as a covariate in all models.

We considered a *P*-value of <0.05 to be statistically significant. To compare groups within months, if the sample month*tourism interaction was significant, we used Bonferonni-adjusted pairwise differences to reduce the risk of inflated error. For each sample month, we included means and standard errors for each of the dependent variables in each possible combination of subspecies, site and sex.

## Results

### Female *C. c. inornata*

There was a significant month*tourist interaction for the suite of physiological dependent variables (F_24, 308_ = 1.99, *P* = 0.004); therefore, we ran separate MANCOVA’s with the same dependent variables within each month (May, June and September). In May, females with follicles had a significantly higher triglyceride index (F_1, 39_ = 30.73, *P* < 0.001; Supplementary Fig. 1a), bactericidal ability (F_1, 39_ = 8.79, *P* = 0.005; Supplementary Fig. 1b), oxidative index (F_1, 39_ = 31.96, *P* < 0.001; Supplementary Fig. 1c) and circulating estradiol concentration (F_1, 39_ = 6.27, *P* = 0.017; Supplementary Fig. 2b). There was significantly higher corticosterone (F_2, 39_ = 5.34, *P* = 0.009; Fig. 3a, Supplementary Table 1) at moderate-tourism sites than either high-tourism (*P* = 0.027) or no-tourism (*P* = 0.031) sites (which were not different from each other, *P* = 0.809). Finally, estradiol differed significantly among sites (F_2, 39_ = 7.72, *P* = 0.001; Supplementary Table 1), with higher estradiol at sites with no tourism (*P* = 0.045) or moderate tourism (*P* = 0.003) than the site with high tourism. There was no significant difference in estradiol between the moderate-tourism and no-tourism sites (*P* = 0.999).

**Figure 1:**
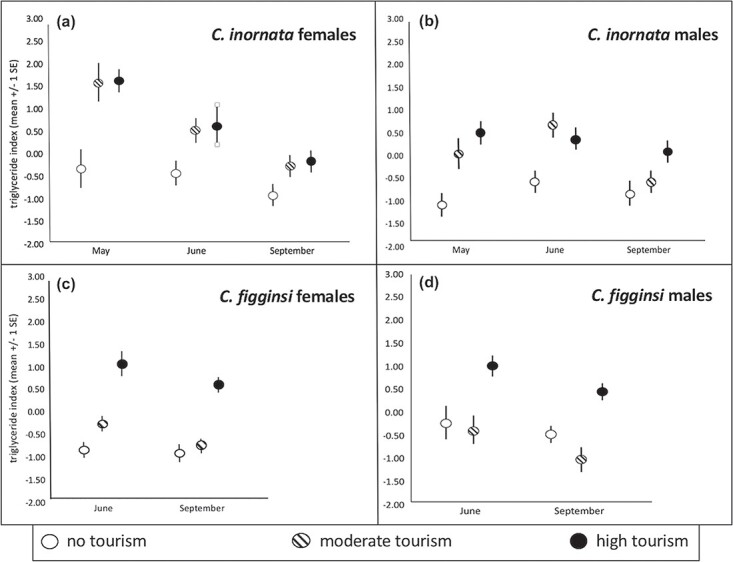
Variation across months in triglyceride index for (**a**) female *C. c. inornata*, (**b**) male *C. c. inornata,* (**c**) female *C. c. figginsi* and (**d**) male *C. c. figginsi* at sites with different levels of tourism.

**Figure 2:**
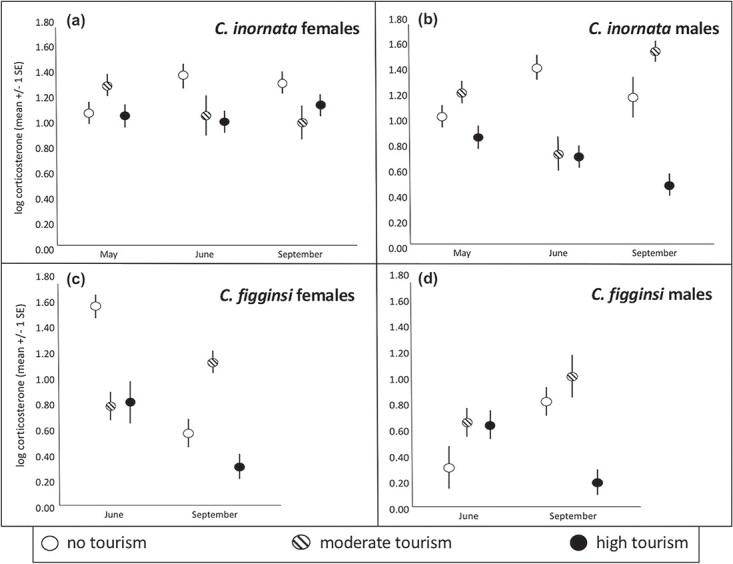
Variation across months in corticosterone concentrations for (**a**) female *C. c. inornata*, (**b**) male *C. c. inornata,* (**c**) female *C. c. figginsi* and (**d**) male *C. c. figginsi* at sites with different levels of tourism.

**Figure 3:**
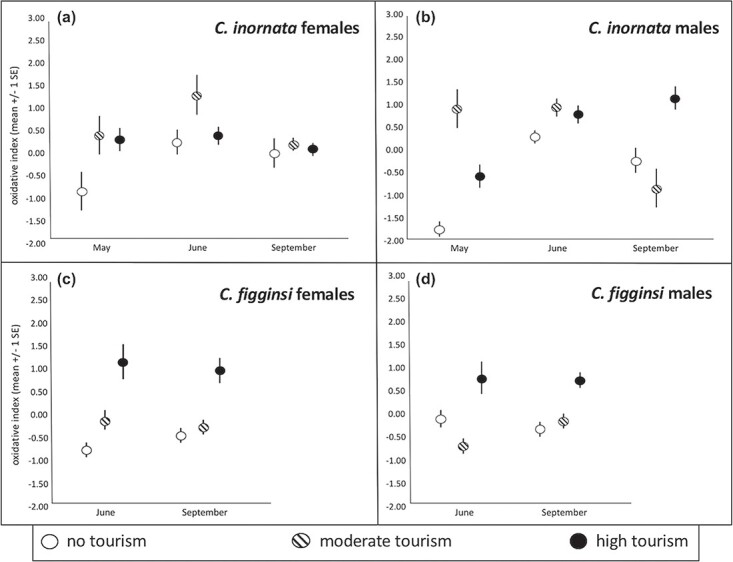
Variation across months in oxidative index for (**a**) female *C. c. inornata*, (**b**) male *C. c. inornata,* (**c**) female *C. c. figginsi* and (**d**) male *C. c. figginsi* at sites with different levels of tourism

In June, the only significant difference among these physiological measures was for progesterone (F_1, 22_ = 9.30, *P* = 0.001), with greater progesterone in females with follicles or eggs (Supplementary Fig. 2a). Among sites with different tourist activity, triglyceride index differed among sites (F_2, 22_ = 7.48, *P* = 0.003; Fig. 1a, Supplementary Table 2). Non-tourist sites had a lower triglyceride index than both moderate- (*P* = 0.003) and high-tourism (*P* = 0.050) sites with no difference between sites with tourism (*P* = 0.900). Corticosterone (F_2, 22_ = 3.49, *P* = 0.048; Fig. 2a, Supplementary Table 2) was significantly greater at sites without tourism than sites with moderate tourism (*P* = 0.025) and sites with high tourism (*P* = 0.024), though there was no difference between the sites with tourism (*P* = 0.999). Finally, oxidative index (F_2, 22_ = 6.86, *P* = 0.005; Fig. 3a, Supplementary Table 2) was greater at the moderate-tourism site than the site without tourism (*P* = 0.009), but there were no other pairwise differences (*P* < 0.089 in both cases).

**Figure 4:**
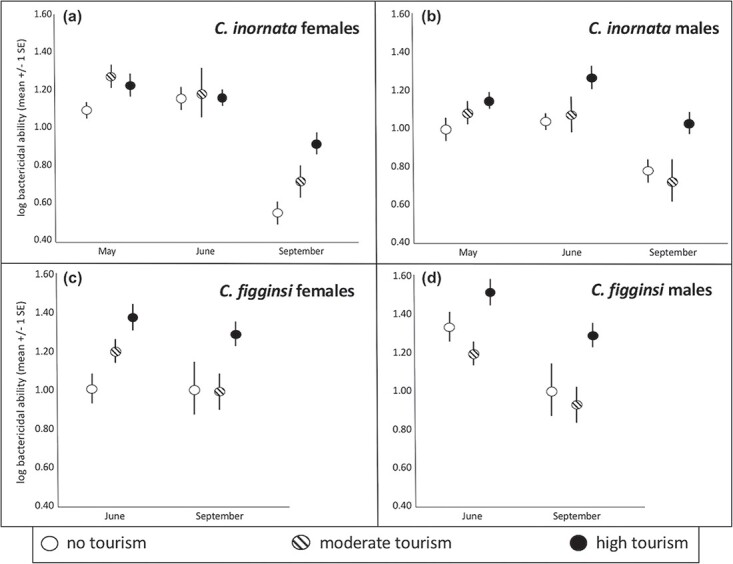
Variation across months in bactericidal ability for (**a**) female *C. c. inornata*, (**b**) male *C. c. inornata,* (**c**) female *C. c. figginsi* and (**d**) male *C. c. figginsi* at sites with different levels of tourism.

In September, only corticosterone differed significantly based on tourism level (F_2, 23_ = 3.33, *P* = 0.048; Fig. 4a, Supplementary Table 3), with significantly greater corticosterone at the site without tourism than the site with moderate tourism (*P* = 0.009). There were no other pairwise differences (*P* > 0.090 in both cases).

Bactericidal ability differed by month independent of tourism (F_2, 93_ = 8.65, *P* < 0.001; Supplementary Tables 1–3), with significantly greater means in May and June compared to September (*P* < 0.001 in both cases), but no difference between May and June (*P* = 0.498). Bactericidal ability also differed by site independent of month (F_2, 93_ = 4.68, *P* = 0.014; Fig. 4a, Supplementary Tables 1–3). Bactericidal ability was significantly greater at the high-tourism site than the site without tourism (*P* = 0.005), with no other pairwise differences (*P* > 0.184 in both cases).

### Female *C. c. figginsi*

There was a significant month*tourist interaction for the suite of physiological dependent variables (F_12, 118_ = 3.12, *P* = 0.001); therefore, we ran separate MANCOVA’s with the same dependent variables within each month (June and September). In June, there were no significant differences in any of the physiological measures between females with follicles and those without follicles (*P* > 0.158 in all cases). Triglyceride index differed among sites based on the level of tourism (F_2,31_ = 16.65, *P* < 0.001; Fig. 1c, Supplementary Table 2), with progressively higher triglyceride index values with increasing tourism (*P* < 0.026 in all cases). There were significant differences among the levels of tourism for corticosterone (F_2, 31_ = 14.26, *P* < 0.001; Fig. 2c, Supplementary Table 2), with higher corticosterone at the site without tourism than both moderate (*P* < 0.001) and high (*P* = 0.001) levels of tourism. There was no significant difference between the sites with tourism (*P* = 0.999). Oxidative index also differed based on the levels of tourism (F_2, 31_ = 15.87, *P* < 0.001; Fig. 3c, Supplementary Table 2), with significantly greater oxidative index at the site with the most tourism compared to sites with moderate tourist activity (*P* < 0.001) and no tourism (*P* < 0.001). The difference between the site without tourism and the site with moderate tourism was not significant (*P* = 0.071).

In September, there were significant differences among the levels of tourism for the same three variables, but the nature of the relationships was different. The triglyceride index differed among sites (F_2, 30_ = 12.82, *P* < 0.001; Fig. 1c, Supplementary Table 3), with the high-tourism site having much higher means than the moderate-tourism (*P* = 0.001) and no-tourism (*P* < 0.001) sites (which were not different from each other, *P* = 0.999). Corticosterone differed among sites (F_2, 30_ = 7.10, *P* = 0.003; Fig. 3b, Supplementary Table 3), with sites experiencing moderate tourist activity having a greater mean value than the site without tourism (*P* = 0.005) and the site with high tourist activity (*P* = 0.043). There was no significant difference between the high-tourism and no-tourism sites (*P* = 0.999). The oxidative index also differed among sites (F_2, 30_ = 7.46, *P* = 0.002; Fig. 3c, Supplementary Table 3) with a higher mean oxidative index at sites with high tourism than the site with moderate tourist activity (*P* = 0.005) and the site with no tourism (*P* = 0.004), with the latter two not significantly different from each other (*P* = 0.999).

Progesterone was not involved in any significant interaction, but progesterone was significantly higher in June than September (F_1, 64_ = 7.11, *P* = 0.01; Supplementary Fig. 3a, Supplementary Tables 2 and 3). Progesterone did not differ significantly among sites with different levels of tourism (F_1,64_ = 1.024, *P* = 0.299, Supplementary Tables 2 and 3). Estradiol was also not involved in any significant interaction, nor did it differ significantly by month (F_1, 62_ = 1.17, *P* = 0.284; Supplementary Fig. 3b, Supplementary Tables 2 and 3) or tourism levels (F_2, 62_ = 0.957, *P* = 0.390; Supplementary Tables 2 and 3).

Bactericidal activity was not involved in any significant interaction and did not differ significantly by month (F_1, 62_ = 2.50, *P* = 0.064; Supplementary Tables 2 and 3). There were significant differences in bactericidal ability among sites with different tourist activity (F_2, 62_ = 4.57, *P* = 0.014; Fig. 4c, Supplementary Tables 2 and 3). Bactericidal ability was significantly greater at the high-tourism site than at the moderate-tourism (*P* = 0.03) and no-tourism (*P* < 0.001) sites (which were not different from each other, *P* = 0.393).

### Male *C. c. inornata*

There was a significant month*tourist interaction for the suite of physiological dependent variables (F_10, 442_ = 5.46, *P* < 0.001); therefore, we ran separate MANCOVA’s with the same dependent variables within each month (May, June and September). In May, triglyceride index differed among sites (F_2,57_ = 25.07, *P* < 0.001; Fig. 1b, Supplementary Table 1), with a higher mean value at sites with high tourism (*P* < 0.001) and moderate tourism (*P* < 0.001) compared to the site with no tourism. There were significantly different corticosterone levels based on the level of tourism (F_2,57_ = 4.33, *P* = 0.018; Fig. 2b, Supplementary Table 1). Iguanas at the moderate-tourism site had significantly greater corticosterone than those at the high-tourism site (*P* = 0.016), but there were no other pairwise differences (*P* > 0.368 in both cases). Oxidative index differed among sites (F_2, 57_ = 17.81, *P* < 0.001; Fig. 3b, Supplementary Table 1), with significantly greater oxidative index at the site with moderate tourism compared to the sites with no tourist activity (*P* < 0.001) and high tourist activity (*P* = 0.002). Further, oxidative index at the site with high tourist activity was also significantly greater than at the site with no tourist activity (*P* = 0.002). Bactericidal ability (F_2, 57_ = 3.26, *P* = 0.046; Fig. 4b, Supplementary Table 1) was significantly greater at the high-tourism site than the site without tourism (*P* = 0.05), but there were no other pairwise differences (*P* > 0.578 in both cases).

In June, triglyceride index differed among sites (F_2,43_ = 29.97, *P* < 0.001; Fig. 1b, Supplementary Table 2), with higher mean values at sites with high tourism (*P* < 0.001) and moderate tourism (*P* < 0.001) compared to the site with no tourism. There was no significant difference in triglycerides between the sites with tourism (*P* = 0.586). However, there were significant differences in corticosterone based on the level of tourism (F_2,43_ = 19.43, *P* < 0.001; Fig. 2b, Supplementary Table 2). Oxidative index differed among sites (F_2, 43_ = 9.24, *P* < 0.001; Fig. 3b, Supplementary Table 2), with significantly greater oxidative indices at the sites with moderate tourism (*P* = 0.001) and high tourism (*P* = 0.002) compared to the site with no tourist activity. There was no significant difference between the sites with tourism (*P* = 0.999).Bactericidal ability (F_2, 43_ = 5.35, *P* = 0.008; Fig. 4b, Supplementary Table 2) was significantly greater at the high-tourism site than at the site without tourism (*P* = 0.009), but there were no other pairwise differences (*P* > 0.132 in both cases). Iguanas at the site without tourism had significantly greater corticosterone than those at the moderate-tourism site (*P* < 0.001) and the high-tourism site (*P* < 0.001), but there was no difference between sites with tourism (*P* = 0.999).

In September, triglyceride index differed among sites (F_2,39_ = 9.09, *P* = 0.001; Fig. 1b, Supplementary Table 3), with a higher mean at the site with high tourism than the site with no tourism (p < 0.001), but there were no other pairwise differences (*P* > 0.150 in both cases). There were significant differences in corticosterone based on the level of tourism (F_2,39_ = 15.19, *P* < 0.001; Fig. 2b, Supplementary Table 3). Iguanas at the site with high tourism had significantly lower corticosterone than those at the sites with moderate tourism (*P* < 0.001) and no tourism (*P* = 0.005), but there was no difference between the moderate-tourism and no-tourism sites (*P* = 0.534). Oxidative index differed among sites (F_2, 39_ = 8.26, *P* = 0.001; Fig. 3b, Supplementary Table 3), with a significantly greater oxidative index at the site with high tourism compared to the sites with moderate tourism (*P* = 0.005) and no tourism (*P* = 0.050). There was no significant difference between the latter two sites (*P* = 0.802). Bactericidal ability (F_2, 39_ = 4.86, *P* = 0.014; Fig. 4b, Supplementary Table 3) was significantly greater at the high-tourism site than both the site without tourism (*P* = 0.050) and the site with moderate tourism (*P* = 0.042), but there was no difference between the latter two sites (*P* = 0.999).

**Figure 5:**
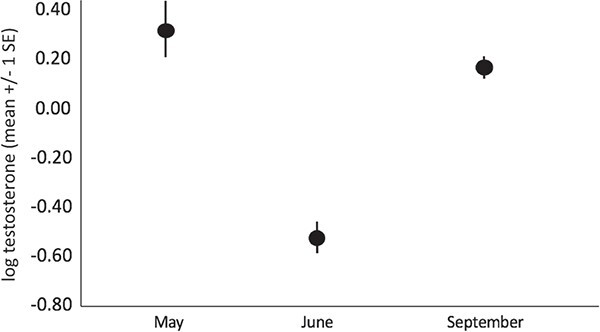
Variation in testosterone for male *C. c. inornata* in different months

Iguanas had significantly lower testosterone (F_2, 137_ = 12.53, *P* < 0.001; Fig. 5, Supplementary Tables 1–3) in June than in May (*P* = 0.011) and September (*P* = 0.006), with no difference between May and September (*P* = 0.428). Testosterone levels did not differ among sites.

### Male *C. c. figginsi*

There was a significant month*tourist interaction for the suite of physiological dependent variables (F_10, 132_ = 1.842, *P* = 0.05); therefore, we ran separate MANCOVA’s with the same dependent variables within each month (June and September). In June, triglyceride index differed among sites (F_2,30_ = 12.55, *P* < 0.001; Fig. 1d, Supplementary Table 2), with a higher mean at the site with high tourism compared to both the moderate-tourism (*P* = 0.043) and no-tourism (*P* = 0.001) sites (which did not differ from each other, *P* = 0.625). There were no significant differences in corticosterone based on the level of tourism in June (F_2,30_ = 2.46, *P* = 0.081; Fig. 2d, Supplementary Table 2). Oxidative index differed among sites (F_2, 30_ = 6.25, *P* = 0.005; Fig. 3d, Supplementary Table 2), with significantly greater oxidative index at the site with high tourism compared to sites with moderate tourist activity (*P* < 0.001) and no tourism (*P* < 0.001). The difference between the site without tourism and the site with moderate tourism was not significant (*P* = 0.996). Bactericidal ability (F_2, 30_ = 9.48, *P* = 0.001; Fig. 4d, Supplementary Table 2) was significantly greater at the high-tourism site than the moderate-tourism site (p < 0.001), but there were no other pairwise differences (*P* > 0.279 in both cases).

**Figure 6:**
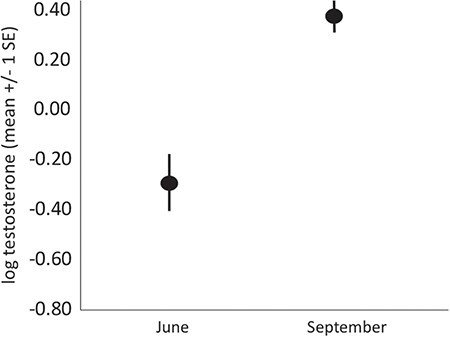
Variation in testosterone for male *C. c. figginsi* in different months

In September, triglyceride index differed among sites (F_2, 39_ = 14.45, *P* < 0.001; Fig. 1d, Supplementary Table 3), with the high-tourism site having much higher means than the moderate-tourism (*P* = 0.001) and no-tourism (*P* < 0.001) sites (which were not different from each other, *P* = 0.329). Corticosterone differed among sites in September (F_2, 39_ = 10.64, *P* < 0.001; Fig. 2d, Supplementary Table 3), with sites with no tourist activity (*P* = 0.002) and the site with moderate tourist activity (*P* < 0.001) having greater means than the site with high tourist activity. There was no significant difference between the moderate-tourism and no-tourism sites (*P* = 0.900). The oxidative index differed among the sites as well (F_2, 39_ = 6.57, *P* = 0.003; Fig. 3d, Supplementary Table 3), with a higher mean oxidative index at the site with high tourism than either the site with moderate tourist activity (*P* = 0.014) or the site with no tourism (*P* = 0.006), with the latter two not significantly different from each other (*P* = 0.999). Bactericidal ability (F_2, 39_ = 6.48, *P* = 0.004; Fig. 4d, Supplementary Table 2) was significantly greater at the high-tourism site than at the moderate-tourism site (*P* = 0.011), but there were no other pairwise differences (*P* > 0.679 in both cases). Lastly, testosterone was not involved in any significant interaction, and levels in September were significantly greater than in June (F_1, 69_ = 23.64, *P* < 0.001; Fig. 6, Supplementary Tables 1-2). Testosterone levels did not differ among sites with different levels of tourism.

## Discussion

### Overview

Overall, we found significant effects of tourism level and season on the physiology of both *C. c. inornata* and *C. c. figginsi*, supporting the idea that tourism is leading to the divergence of phenotypes. In general, we found elevated plasma energy metabolites, oxidative stress and bactericidal ability, but reduced corticosterone concentrations with increasing tourism in both subspecies of rock iguanas. We also found that season and tourism level interacted significantly such that they are inextricable linked. Despite variation with season (and reproductive status), the physiological metrics corresponded to tourism level in both subspecies and persist, to some extent, across seasons. These results suggest that anthropogenic disturbance is resulting in disparate physiologies in northern Bahamian rock iguanas, but more work is required to test the consistency of these changes over time.

### Tourism-induced phenotypes

The primary tourism effects manifested predominantly in two ways: (i) some physiological metrics corresponded to tourism level in a step-wise fashion such that there were clear differences among high, moderate and no-tourism populations; or (ii) other metrics had an effect such that high- and no-tourism sites differed, but moderate sites were more similar to either high- or no-tourism sites. We also found some variation in physiological measures among sexes, subspecies and seasons. Specifically, *C. c. figginsi* of both sexes at the high-tourism site had higher concentrations of energy metabolites (i.e. an index of triglycerides and free glycerol) than moderate- and no-tourism sites, and all fed populations (both moderate and high) had elevated energy metabolites in *C. c. inornata* (except during September when only high-tourist iguanas had elevated metabolites relative to other populations). These findings are not surprising given that supplemental feeding of many types of wildlife by tourists is a large part of the tourism industry in The Bahamas and elsewhere (Shannon *et al.,* 2017). Supplementation with high-calorie foods has been shown to lead to elevated levels of blood glucose and lipids (Knapp *et al.,* 2013), which could alter metabolic and physiological processes, such as immune function, dependent on nutritional resources (Semeniuk *et al.,* 2009; Strandin *et al.,* 2018).

Interestingly in the present study, our measure of innate immunity, bacterial killing ability, was generally highest for male and female iguanas at high-tourism sites. Previous work has also shown that consuming unnatural food sources can also have negative impacts on animal health, causing injury and disease (several species of large mammals) (Orams, 2002), increased metabolic rate (whitetip reef sharks, *Triaenodon obesus*) (Barnett *et al.,* 2016) and altered faeces consistency (rock iguanas; Knapp *et al.,* 2013). For example, Galapagos marine iguanas (*Amblyrhynchus cristatus*) exposed to tourism in the absence of food provisioning show reduced immune function (French *et al.,* 2017; French *et al.,* 2010). The apparent elevation in immunity in rock iguanas could therefore be because they acquire additional nutritional resources through feeding that can be allocated to the immune system. This hypothesis is further supported by the relatively higher levels of energy metabolites at high-tourist sites. Additionally, the enhanced innate immunity may result, at least in part, from the unnaturally high densities of rock iguanas on feeding beaches, which could cause an increased transmission of infection or parasites leading to the upregulation of immune activity. For example, fed iguanas at these sites have more incidences of faecal parasites (Knapp *et al.,* 2013), but a relationship between this and increased bacterial killing ability has not yet been established.

Easy and concentrated access to introduced food can lead to unnatural aggregation at feeding sites, which can lead to higher pathogen transmission (avian populations) (Robb *et al.,* 2008), and increased intraspecific and interspecific aggression (sicklefin lemon sharks, *Negaprion acutidens*) (Clua *et al.,* 2010), all of which can result in stress to animals. Unexpectedly, in the present study corticosterone was generally lower for iguanas at sites with more tourist activity, but this varied depending on sex and season for both subspecies. In female *C. c. figginsi* during the nesting season (June), corticosterone was significantly lower at both high- and moderate-tourist sites than at the site with no tourists, but in September when all animals were post-reproductive, corticosterone levels in animals at the moderate-tourist site were elevated above the other sites. Similarly, for *C. c. inornata* populations, corticosterone was lower at high and moderate sites across seasons, except for early reproduction when females from the moderate-tourist site had elevated corticosterone relative to the other populations. The most likely explanation is that environmental variation between the northern and southern cay clusters is leading to differences in reproductive timing and thus physiology. Previous work has shown that nesting dates can vary by up to a week across these cays, even though they are in close proximity (Iverson *et al.,* 2004b). Thus, tourist-induced effects are likely interacting with natural differences in reproduction to affect corticosterone levels over time (Moore and Jessop, 2003). Likewise, corticosterone was consistently lowest in male *C. c. inornata* from the high-tourist site and highest at the no-tourist site. However, corticosterone from the moderate tourist site fluctuated significantly across the season (i.e. sometimes resembling high tourist and sometimes no-tourist sites). Instead, during nesting season, *C. c. figginsi* males had higher corticosterone at high- and moderate-tourist relative to no-tourist sites, but during post-reproduction the corticosterone from high-tourist iguanas dropped significantly and corticosterone in male iguanas from no-tourist sites increased significantly (i.e. similar to males from the moderate sites).

Similarly, French *et al.* (2017) also found that corticosterone varied significantly with tourism intensity in reproductive Galapagos marine Iguanas, but the direction differed between the sexes, whereby baseline corticosterone levels in male iguanas increased and in female iguanas decreased with tourism intensity. However, there was also significant variation over time and depending on specific population and context. For example, other studies with iguanas found no difference in baseline corticosterone concentrations associated with tourism (Galapagos marine Iguanas and northern Bahamian rock iguanas) (Romero and Wikelski, 2002; Knapp *et al.,* 2013). Yet, changes in reactive corticosterone (i.e. corticosterone increases in response to stress) has repeatedly been linked to tourism, albeit in conflicting directions. For example, post-stress corticosterone levels in Galapagos marine iguanas were higher for no-tourism groups in one study (Romero and Wikelski, 2002) and higher only for non-breeding tourist-exposed iguanas in another study (French *et al.,* 2010). This further demonstrates the importance of assessing reproductive condition when trying to understand the physiological implications of external challenges that result in stress (French *et al.,* 2010). In the context of corticosterone, it is also important to note that Galapagos marine guanas are not fed by tourists, in contrast to The Bahamian rock iguanas that have been studied. Corticosterone plays a critical role in mobilizing energy stores and increases when some reptiles face periods of food deprivation compared to when recently fed (Webb *et al.,* 2017). Therefore, in the current study, corticosterone may be lower in rock iguanas experiencing heavy feeding simply because resources are in such abundance. This idea is supported by the significantly greater energy metabolite levels found at fed tourist sites. Alternatively, but not mutually exclusive, the rock iguanas experience such heavy exposure to tourists that they may have habituated to humans, which continuously reinforces iguana aggregations on fed beaches. However, given the relationships observed between human presence and baseline corticosterone concentrations for other iguana species (Romero and Wikelski, 2002; French *et al.,* 2010), the former explanation is more strongly supported.

In general, we found that iguanas at high-tourism sites had higher oxidative indices (i.e. relative amount of reactive oxygen species compared to antioxidants), suggesting that these iguanas may be experiencing a state of oxidative stress, such that their levels of antioxidants may not be sufficient to prevent harm from the reactive oxygen species being produced (Vassalle *et al.,* 2008). Specifically, in *C. c. figginsi* we found that oxidative index in females differed in a step-wise fashion with tourism across seasons, whereby the highest levels were found in females at the high-tourist site and lowest at the no-tourist site. In males of this subspecies the impact was similar but less distinct with significant elevations in oxidative index at high-tourist site, but there was no difference between the other site types. In *C. c. inornata*, males had consistently elevated oxidative indices at both tourist sites (both high and moderate) over the season, except during post-breeding when the oxidative index was only elevated in iguanas at the high-tourist site. Female *C. c. inornata* showed more seasonal fluctuations such that they had elevated oxidative indices at all tourist sites (high and moderate) in early reproduction, but only females from the moderate-tourist site had an elevated oxidative index during nesting and no differences among populations were present during post-reproduction. Interestingly, Galapagos marine iguanas also had higher d-ROMs at high-tourism sites and they were related to tourism intensity across sites in a dose-dependent manner. Given that marine iguanas, unlike rock iguanas, are not fed by tourists yet exhibited relatively similar patterns in both systems, this suggests that the relationship between tourism and oxidative stress could be independent from the feeding experienced by rock iguanas. However, absorptive states of digestion were associated with increased d-ROMs in corn snakes (*Pantherophis guttatus*) (Butler *et al.,* 2016), which could also explain, at least in part, the elevated levels for iguanas experiencing heavy, regular food provisioning. While we are seeing overall patterns in physiology that are largely replicable among the subspecies, we cannot isolate the impacts of tourism from reproduction and season as they are inextricably connected.

### Reproductive and seasonal fluctuations

Although there were significant impacts of tourism on physiology over time, many of these effects were compounded by reproduction (i.e. not all females breed annually) and the reproductive cycle. Reproduction significantly alters iguana physiology (Webb *et al.,* 2019) and can affect individual responses to tourism (French *et al.,* 2010). Moreover, known differences in phenology among the subspecies also contribute to this variation in response. Specifically, *C. c. figginsi* apparently begins its nesting season 1–2 weeks earlier than *C. c. inornata* (Iverson *et al.,* 2004b), which can lead to the manifestation of seasonal differences in physiology and condition. In addition to breeding status, seasonal changes in physiology were also observed, albeit seasonal and reproductive cycle changes are difficult to disentangle. Seasonal physiological fluctuations not surprisingly differed between the sexes, as they experience different reproductive costs (Tilbrook *et al.,* 2002). It is important to highlight that our sampling times coincided with mating and vitellogenesis (May, *C. c. inornata* subspecies only), gravidity and nesting (June, both subspecies) and post-reproductive season (September, both subspecies). Therefore, it is not surprising that we found fluctuations in steroid hormones that correspond to the reproductive process, such as higher estradiol early in reproduction during vitellogenesis and higher progesterone during gravidity and right before and during nesting. Similar fluctuations are seen across species (Weiss *et al.,* 2002; Barbosa-Moyano *et al.,* 2020; Hudson *et al.,* 2020). Interestingly, we observed slight elevations in testosterone in the fall during post-reproduction relative to late reproduction in males of both subspecies. This may be a preparatory response for the subsequent breeding season, although, previous work in closely related species *C. carinata* did not show evidence of increased testes size during this non-breeding time of year (Iverson, 1979). We see a similar September elevation of estradiol levels in female *C. c. figginsi.* Corticosterone, which is also known to change with reproduction and season (Kenagy and Place, 2000; Moore and Jessop, 2003; Lind *et al.,* 2018), appears to fluctuate with season in the current study, but the patterns do not appear to be consistent among sexes or subspecies.

In general, all other physiological metrics in this study are elevated during the reproductive season (both early and late) and then decrease during post breeding, including energy metabolites, bactericidal ability and the oxidative stress index. Past research has shown that both oxidative status and immunity shift with reproductive state and investment in a number of vertebrates, albeit in context-dependent ways (Metcalfe and Monaghan, 2013; Stahlschmidt *et al.,* 2015; Costantini *et al.,* 2016; Schoenle *et al.,* 2017; Webb *et al.,* 2019). For example, female *C. c. inornata* that yolk more follicles show elevated levels of energy metabolites in the blood and also elevated reactive oxygen metabolites (Webb *et al.,* 2019). However, it should also be noted that yolk precursors including vitellogenin may also serve as antioxidants to protect against oxidative damage during reproduction (Costantini *et al.,* 2016; Lindsay *et al.,* 2020). Similarly, immune function is also impacted by reproductive state and investment in many species (Ardia *et al.,* 2003; French *et al.,* 2007; Hayward *et al.,* 2019). At the same time, hormones that mediate responses to stress (i.e. glucocorticoids) can vary according to reproductive state (Woodley and Moore, 2002) and can also impact oxidative status and immunity, albeit the relationship is not necessarily direct and varies significantly with context and duration (Dhabhar, 2009a; Dhabhar, 2009b; Costantini *et al.,* 2011; Spiers *et al.,* 2015).

It is also important to acknowledge that environmental differences including tourism schedules may also vary with season. For example, our post-reproductive study coincided with the hurricane season, which may limit private boat access, and so disproportionately impact moderate-tourism sites that are not visited by large tourist companies, which provide day trips to the sites from major ports and continue year-round. Our ongoing studies evaluating the effects of the sharp drop in tourism due to the coronavirus in 2020–21 may be able to address these potential differences (Today, 2020; Economics, 2021).

### Subspecies considerations

We found the same relative physiological relationships with tourism in both subspecies, despite differences in phenology (*C. c. figginsi* breed 1–2 weeks sooner), and potential inherent differences in natural history. For example, *C. c. inornata* is the northernmost member of its genus and sexual maturity in females requires 12 years (the longest time to maturity in any lizard; Iverson *et al.*, 2004b). The more southern populations of *C. c. figginsi* are less affected by winter cold fronts and apparently mature in fewer years (Knapp, pers. comm). The slight variations in seasonal and tourist-based patterns between subspecies in body condition (Tables 1–3) and physiology are likely a reflection of both genetic (Colosimo *et al.,* 2021) and site (i.e. environmental) differences between northern and southern populations. Moreover, there are known differences in parasitism between *C. c. inornata* and *C. c. figginsi*, whereby *C. c. figginsi* is parasitized by ticks of the genus *Amblyomma* while *C. c. inornata* is not (Durden and Knapp, 2005; Durden *et al.,* 2015). Although past work failed to find significant effects of tick parasite load on annual adult growth rate, corticosterone levels and some haematological parameters (i.e. packed cell volume, total white blood cells, heterophils, monocytes, eosinophils, or haemoglobin), there were significant associations of tick burden with body condition and other immune cells including lymphocyte and basophil counts as well as heterophil-to-lymphocyte ratios (Knapp *et al.,* 2019). These associations varied by iguana sex, size and blood parasite infection status suggesting that different iguana life stages may invest differently in immunity and that the impacts on ectoparasite infection may be modulated based on size and sex of hosts and coinfection status (Knapp *et al.,* 2019). Thus, the effects of parasitic infection may lead to some of the minor deviations in physiology we observed, in particular, body condition and immune measures, among the northern and southern populations.

Additionally, the northern cays have historically been fed at high rates for longer, and thus we may be observing time-course differences in population density effects of tourism (Iverson *et al.,* 2006), whereby the more recent intense tourism effects in the southern cays could eventually be equivalent with the north (Knapp, personal observations). There are also slight differences in tourism pressure and feeding frequency, with tourist-visited northern populations experiencing a slightly higher rate of tourism than tourist-visited southern populations (average of 145 tourist/day versus 119 tourist/day in June) but *C. c. figginsi* experiences tourist visits for longer periods throughout the day (unpublished data).

## Conclusions

Overall, we found generally consistent physiological responses to tourism across populations of endangered iguanas. These new findings coupled with previous work showing differences in blood chemistry (Knapp *et al.,* 2013), behaviour (Hines, 2011), growth and condition (Iverson *et al.,* 2006; Smith and Iverson, 2016) and survival (Iverson *et al.,* 2006) all demonstrate that anthropogenic disturbance leads to disparate physiologies akin to those observed in response to natural variations in the environment. Although, for formal ecotypes to emerge, these responses must be heritable and thus persist over generations, the framework of divergent selective pressures seems to be in place. Moreover, preliminary evidence suggests very little gene flow between cays in close proximity, increasing the likelihood of continued divergence of physiological phenotypes in the future (Malone *et al.,* 2003; Aplasca *et al.,* 2016). In general, high tourism resulted in elevated energy metabolites, immunity and oxidative stress, but decreased levels of corticosterone. In several cases, these relationships correspond with tourism level (i.e. not just presence or absence of tourism). There are also important interactions with reproductive state and season that must be considered, as well as whether these effects are consistent over time.

Future work should focus on the mechanisms linking these physiological changes to fitness and survival in these endangered iguanas. For example, access to more food at tourist sites has been shown to alter reproductive rates and timing in females where food is often a limiting factor (black bears) (Dunkley and Cattet, 2003; Robb *et al.,* 2008) and an increase in reproduction could further alter female health (rock iguanas) by increasing exposure to oxidative stress (Webb *et al.,* 2019), potentially affecting survival. However, more work will need to look at long-term survival trajectories and to integrate reproductive rates and potential physiological costs into population models to investigate these possible mechanisms (Ames *et al.,* 2020).

## Funding

This work was supported by Utah State University Research Catalyst Grant [grant number A35851 to S.S.F.], Utah Agricultural Experiment Station [project numbers 1104 and 1347 to S.S.F.], National Science Foundation [grant number (IOS)-1752908 to S.S.F.], Utah State University Ecology Center [to A.C.W.], Shedd Aquarium [to C.R.K.], Dr Scholl Foundation [to C.R.K.], Paul M. Angell Family Foundation [to C.R.K], the Earlham College Biology Department [to J.B.I.] and the personal funds of most of the field assistants.

## Data Accessibility

Data related to published articles will be available on USU Digital Commons.

## Supplementary Material

suppl_data_coac001
