# Supplementary material for: Complex tourism and season interactions contribute to disparate physiologies in an endangered rock iguana
Source: Conserv Physiol. 2022 Feb 5;10(1):coac001. doi: 10.1093/conphys/coac001 (PMC9040281; doi:10.1093/conphys/coac001)
Supplement: suppl_data_coac001 [file suppl_data_coac001.zip › Supplementary Material.docx]

**Supplementary Material**

**
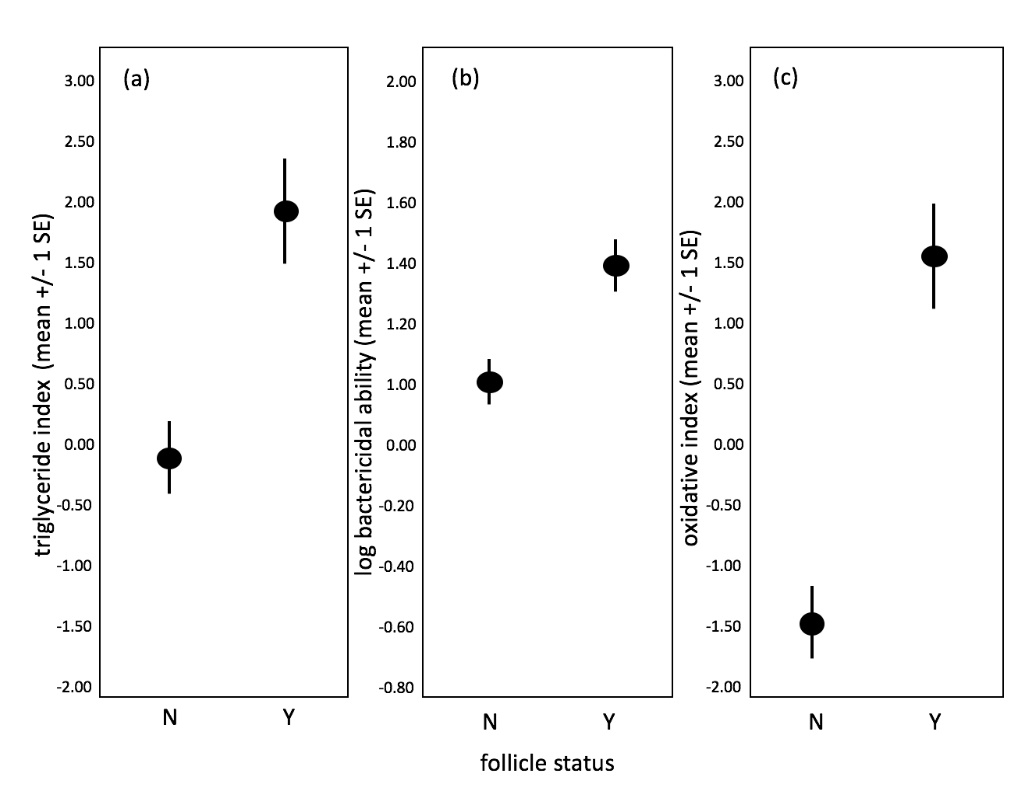
**

**Supplementary Figure 1a-c. Physiological differences in triglyceride index (a), bactericidal ability (b), and oxidative index (c) for female *C. c. inornata* without (N) and with (Y) developing ovarian follicles in May.**

**
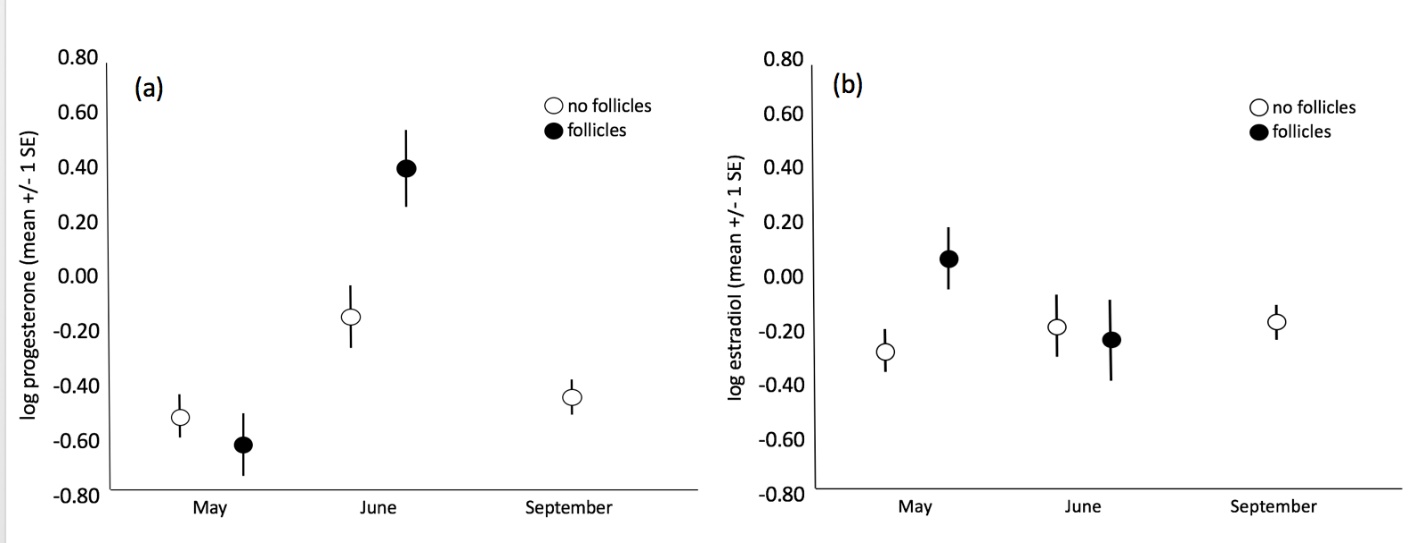
**

**Supplementary Figure 2a-b. Variation across months in progesterone (a) and estradiol (b) for female *C. c. inornata* without (open circles) and with (filled circles) developing ovarian follicles.**

**
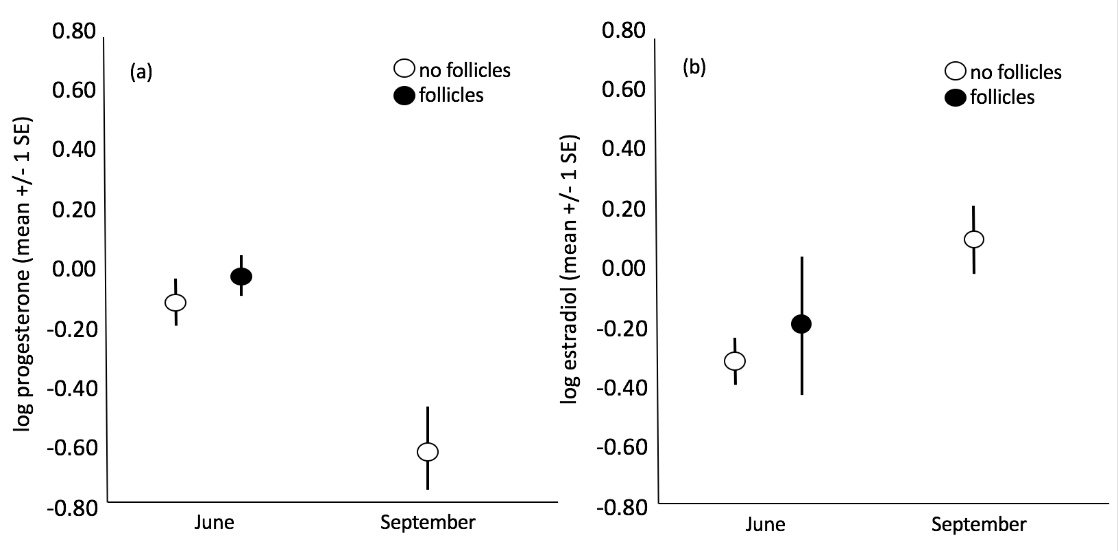
**

**Supplementary Figure 3a-b. Differences in progesterone (a) and estradiol (b) for female *C. c. figginsi* with and without ovarian follicles in different months.**

**Supplementary Tables 1-6: Descriptive tables (means +/- SE)**

**Table 1: May**

| Subspecies | Site (Tourism) | Sex | n | Prog (ng/mL) | E_2_ (ng/mL) | T (ng/mL) | CORT(ng/mL) | TriIndex | OxyIndex | BKA (%) | BCI |
| --- | --- | --- | --- | --- | --- | --- | --- | --- | --- | --- | --- |
| *inornata* | Flat Rock Reef Cay (No) | F | 13 | 0.38 ± 0.09 | 1.32 ± 0.41 | -- | 14.49 ± 2.99 | -0.23 ± 0.39 | -0.90 ± 0.52 | 14.47 ± 2.48 | 29.83 ± 3.79 |
|  |  | M | 17 | -- | -- | 5.86 ± 2.18 | 12.03 ± 1.41 | -1.06 ± 0.13 | -2.19 ± 0.12 | 13.39 ± 3.37 | 40.66 ± 3.27 |
|  | U Cay (Low) | F | 21 | 0.33 ± 0.11 | 1.45 ± 0.17 | -- | 18.73 ± 1.77 | 1.41 ± 0.36 | 0.21 ± 0.47 | 28.00 ± 4.67 | 26.23 ± 2.94 |
|  |  | M | 14 | -- | -- | 15.35 ± 2.41 | 18.51 ± 3.03 | 0.09 ± 0.26 | 0.73 ± 0.33 | 16.16 ± 2.97 | 41.56 ± 3.60 |
|  | Allens Leaf Cay (High) | F | 29 | 0.40 ± 0.07 | 0.70 ± 0.14 | -- | 12.17 ± 0.90 | 1.45 ± 0.22 | 0.12 ± 0.34 | 27.06 ± 3.44 | 30.73 ± 2.50 |
|  |  | M | 39 | -- | -- | 11.97 ± 1.48 | 9.77 ± 0.74 | 0.43 ± 0.10 | -0.76 ± 0.25 | 16.60 ± 1.99 | 38.86 ± 2.19 |

| Subspecies | Site (Tourism) | Sex | n | Prog (ng/mL) | E_2_ (ng/mL) | T (ng/mL) | CORT (ng/mL) | TriIndex | OxyIndex | BKA (%) | BCI |
| --- | --- | --- | --- | --- | --- | --- | --- | --- | --- | --- | --- |
| *figginsi* | Noddy Cay (No) | F | 16 | 1.04 ± 0.10 | 1.52 ± 0.30 | -- | 46.77 ± 4.43 | -0.77 ± 0.13 | -0.56 ± 0.13 | 12.90 ± 2.76 | 27.43 ± 2.04 |
|  |  | M | 7 | -- | -- | 0.13 ± 1.39 | 3.94 ± 4.34 | -0.08 ± 0.28 | 0.03 ± 0.21 | 22.85 ± 5.53 | 47.59 ± 3.08 |
|  | White Bay Cay (Low) | F | 19 | 0.80 ± 0.09 | 0.73 ± 0.22 | -- | 15.40 ± 4.07 | -0.27 ± 0.12 | -024 ± 0.12 | 19.16 ± 2.54 | 28.13 ± 1.87 |
|  |  | M | 18 | -- | -- | 1.00 ± 0.89 | 6.18 ± 2.71 | -0.27 ± 0.15 | -0.30 ± 0.12 | 17.69 ± 3.55 | 41.45 ± 2.04 |
|  | Leaf Cay (High) | F | 8 | 0.87 ± 0.16 | 0.90 ± 0.32 | -- | 9.27 ± 7.24 | 0.97 ± 0.20 | 0.65 ± 0.20 | 27.99 ± 3.91 | 32.94 ± 2.88 |
|  |  | M | 17 | -- | -- | 2.92 ± 0.89 | 5.40 ± 2.97 | 0.92 ± 0.15 | 0.51 ± 0.13 | 36.13 ± 3.78 | 50.59 ± 2.10 |
| *inornata* | Flat Rock Reef Cay (No) | F | 13 | 0.91 ± 0.22 | 0.71 ± 0.20 | -- | 27.05 ± 4.21 | -0.36 ± 0.28 | 0.11 ± 0.40 | 17.90 ± 4.10 | 34.16 ± 3.86 |
|  |  | M | 15 | -- | -- | 0.36 ± 2.32 | 28.82 ± 2.32 | -0.64 ± 0.16 | 0.02 ± 0.29 | 12.97 ± 3.56 | 39.79 ± 3.58 |
|  | U Cay (Low) | F | 5 | 1.50 ± 0.31 | 0.97 ± 0.34 | -- | 21.13 ± 3.34 | 0.49 ± 0.39 | 1.23 ± 0.56 | 27.91 ± 5.79 | 34.58 ± 5.99 |
|  |  | M | 14 | -- | -- | 0.75 ± 2.41 | 8.89 ± 2.68 | 0.54 ± 0.17 | 0.66 ± 0.29 | 25.26 ± 3.68 | 31.99 ± 3.58 |
|  | Allens Leaf Cay (High) | F | 17 | 1.51 ± 0.19 | 0.84 ± 0.16 | -- | 13.74 ± 3.53 | 0.38 ± 0.24 | 0.18 ± 0.34 | 17.63 ± 3.44 | 26.29 ± 3.25 |
|  |  | M | 22 | -- | -- | 3.04 ± 1.96 | 7.70 ± 2.24 | 0.35 ± 0.13 | 0.51 ± 0.23 | 27.80 ± 2.94 | 32.08 ± 2.92 |

**Table 2: June**

**Table 3: September**

| Subspecies | Site (Tourism) | Sex | n | Prog (ng/mL) | E_2_ (ng/mL) | T (ng/mL) | CORT (ng/mL) | TriIndex | OxyIndex | BKA (%) | BCI |
| --- | --- | --- | --- | --- | --- | --- | --- | --- | --- | --- | --- |
| *figginsi* | Noddy Cay (No) | F | 10 | 0.52 ± 0.13 | 0.94 ± 0.30 | -- | 6.28 ± 5.61 | -0.86 ± 0.17 | 0.02 ± 0.17 | 12.71 ± 3.50 | 30.60 ± 3.58 |
|  |  | M | 18 | -- | -- | 4.79 ± 0.87 | 8.67 ± 2.71 | -0.31 ± 0.15 | 0.48 ± 0.14 | 18.99 ± 3.45 | 39.13 ± 2.75 |
|  | White Bay Cay (Low) | F | 15 | 0.33 ± 0.11 | 1.40 ± 0.22 | -- | 19.62 ± 4.74 | -0.70 ± 0.14 | 0.11 ± 0.14 | 11.55 ± 2.86 | 29.08 ± 2.92 |
|  |  | M | 14 | -- | -- | 3.30 ± 0.99 | 17.33 ± 3.07 | -0.76 ± 0.17 | 0.01 ± 0.14 | 10.82 ± 3.91 | 36.91 ± 3.03 |
|  | Leaf Cay (High) | F | 17 | 0.56 ± 0.10 | 1.54 ± 0.21 | -- | 4.45 ± 4.30 | 0.68 ± 0.13 | 0.89 ± 0.13 | 23.51 ± 2.68 | 43.06 ± 2.75 |
|  |  | M | 13 | -- | -- | 4.30 ± 1.02 | 2.75 ± 3.19 | 0.48 ± 0.15 | 0.48 ± 0.14 | 22.11 ± 4.06 | 61.56 ± 3.27 |
| *inornata* | Flat Rock Reef Cay (No) | F | 10 | 0.64 ± 0.24 | 0.59 ± 0.22 | -- | 28.87 ± 4.61 | -0.99 ± 0.34 | -0.10 ± 0.46 | 3.53 ± 4.49 | 28.23 ± 4.02 |
|  |  | M | 13 | -- | -- | 1.50 ± 2.49 | 21.39 ± 2.90 | -0.91 ± 0.18 | -0.55 ± 0.30 | 6.47 ± 3.82 | 49.61 ± 3.53 |
|  | U Cay (Low) | F | 17 | 0.46 ± 0.18 | 0.83 ± 0.21 | -- | 29.66 ± 3.64 | -0.65 ± 0.23 | 0.16 ± 0.34 | 4.95 ± 3.44 | 29.98 ± 3.08 |
|  |  | M | 13 | -- | -- | 2.58 ± 2.49 | 30.44 ± 2.78 | -0.78 ± 0.17 | -1.19 ± 0.33 | 5.58 ± 3.82 | 34.61 ± 3.53 |
|  | Allens Leaf Cay (High) | F | 17 | 0.61 ± 0.19 | 0.74 ± 0.17 | -- | 12.93 ± 3.53 | -0.64 ± 0.23 | -0.01 ± 0.34 | 9.70 ± 3.44 | 26.44 ± 3.08 |
|  |  | M | 22 | -- | -- | 2.61 ± 1.92 | 6.16 ± 2.14 | -0.08 ± 0.13 | 0.70 ± 0.23 | 12.56 ± 2.94 | 37.61 ± 2.71 |

**Table 4: May size data**

| Subspecies | Site (Tourism) | Sex | n | Body mass | SVL |
| --- | --- | --- | --- | --- | --- |
| *inornata* | Flat Rock Reef Cay (No) | F | 13 | 806.38 ± 155.04 | 26.49 ± 1.41 |
|  |  | M | 17 | 1384.00 ± 135.58 | 31.88 ± 1.23 |
|  | U Cay (Low) | F | 21 | 745.60 ± 125.00 | 26.02 ± 1.11 |
|  |  | M | 14 | 1420.36 ± 149.40 | 30.66 ± 1.35 |
|  | Allens Leaf Cay (High) | F | 29 | 786.45 ± 103.80 | 26.80 ± 0.94 |
|  |  | M | 39 | 1262.84 ± 90.68 | 31.27 ± 0.82 |

**Table 5: June size data**

| Subspecies | Site (Tourism) | Sex | n | Body mass | SVL |
| --- | --- | --- | --- | --- | --- |
| *figginsi* | Noddy Cay (No) | F | 16 | 737.19 ± 132.87 | 26.70 ± 1.12 |
|  |  | M | 7 | 1522.14 ± 200.88 | 31.79 ± 1.69 |
|  | White Bay Cay (Low) | F | 19 | 786.32 ± 121.93 | 27.75 ± 1.03 |
|  |  | M | 18 | 1296.25 ± 132.87 | 31.03 ± 1.12 |
|  | Leaf Cay (High) | F | 8 | 988.75 ± 187.91 | 29.19 ± 1.58 |
|  |  | M | 17 | 1819.33 ± 137.23 | 34.44 ± 1.16 |
| *inornata* | Flat Rock Reef Cay (No) | F | 13 | 1093.64 ± 160.25 | 29.93 ± 1.35 |
|  |  | M | 15 | 1383.93 ± 142.05 | 32.52 ± 1.19 |
|  | U Cay (Low) | F | 5 | 1114.17 ± 216.98 | 31.58 ± 1.83 |
|  |  | M | 14 | 993.00 ± 142.05 | 27.34 ± 1.19 |
|  | Allens Leaf Cay (High) | F | 17 | 729.41 ± 128.91 | 27.27 ± 1.09 |
|  |  | M | 22 | 990.14 ± 115.98 | 30.01 ± 0.96 |

**Table 6: September size data**

| Subspecies | Site (Tourism) | Sex | n | Body mass | SVL |
| --- | --- | --- | --- | --- | --- |
| *figginsi* | Noddy Cay (No) | F | 10 | 869.5 ± 185.18 | 28.04 ± 1.46 |
|  |  | M | 18 | 1193.61 ± 138.03 | 29.31 ± 1.12 |
|  | White Bay Cay (Low) | F | 15 | 843.00 ± 151.20 | 28.69 ± 1.19 |
|  |  | M | 14 | 1139.64 ± 156.51 | 30.20 ± 1.23 |
|  | Leaf Cay (High) | F | 17 | 1371.12 ± 142.03 | 30.79 ± 1.12 |
|  |  | M | 13 | 2324.58 ± 169.05 | 36.52 ± 1.28 |
| *inornata* | Flat Rock Reef Cay (No) | F | 10 | 781.500 ± 185.18 | 25.94 ± 1.46 |
|  |  | M | 13 | 1825.00 ± 162.41 | 33.48 ± 1.28 |
|  | U Cay (Low) | F | 17 | 871.47 ± 142.03 | 28.31 ± 1.12 |
|  |  | M | 13 | 1105.00 ± 162.41 | 29.25 ± 1.28 |
|  | Allens Leaf Cay (High) | F | 17 | 727.94 ± 142.03 | 27.31 ± 1.12 |
|  |  | M | 22 | 1202.96 ± 124.85 | 30.76 ± 0.98 |
