# Supplementary figures and images for: Complex tourism and season interactions contribute to disparate physiologies in an endangered rock iguana
Source: Conserv Physiol. 2022 Feb 5;10(1):coac001. doi: 10.1093/conphys/coac001 (PMC9040281; doi:10.1093/conphys/coac001)

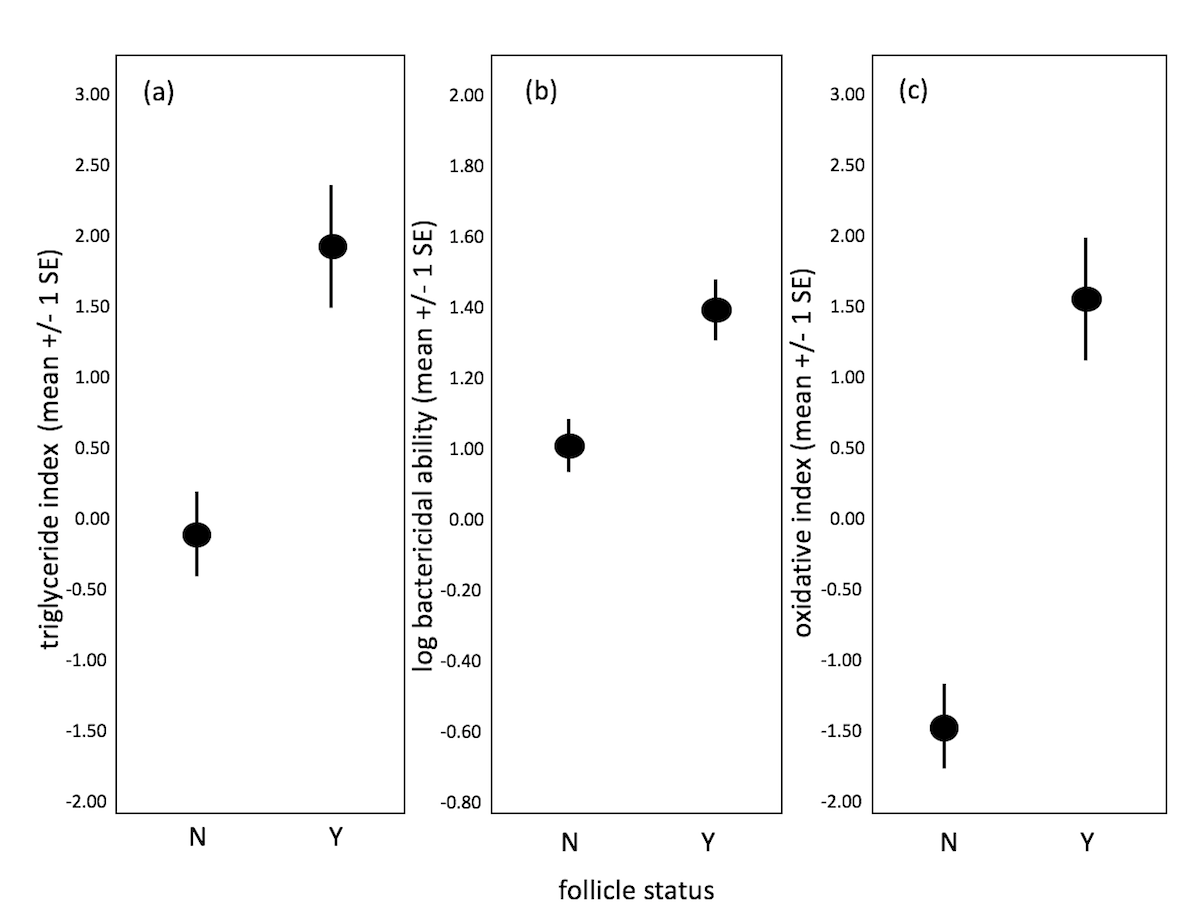

Supplement: suppl_data_coac001 [file suppl_data_coac001.zip › IguanaSupFig1_UPDATE.tiff]

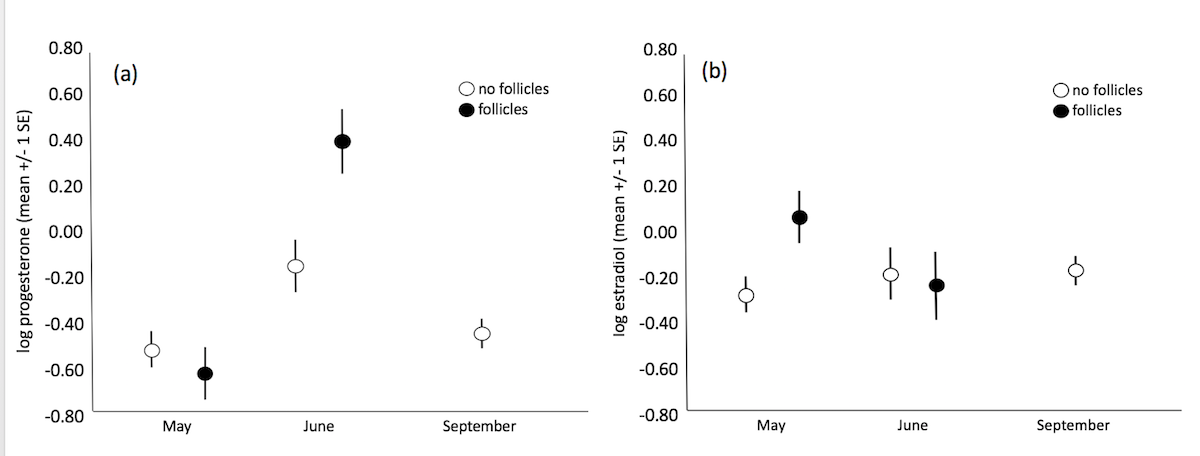

Supplement: suppl_data_coac001 [file suppl_data_coac001.zip › Iguana_SupFig2_Update.tiff]

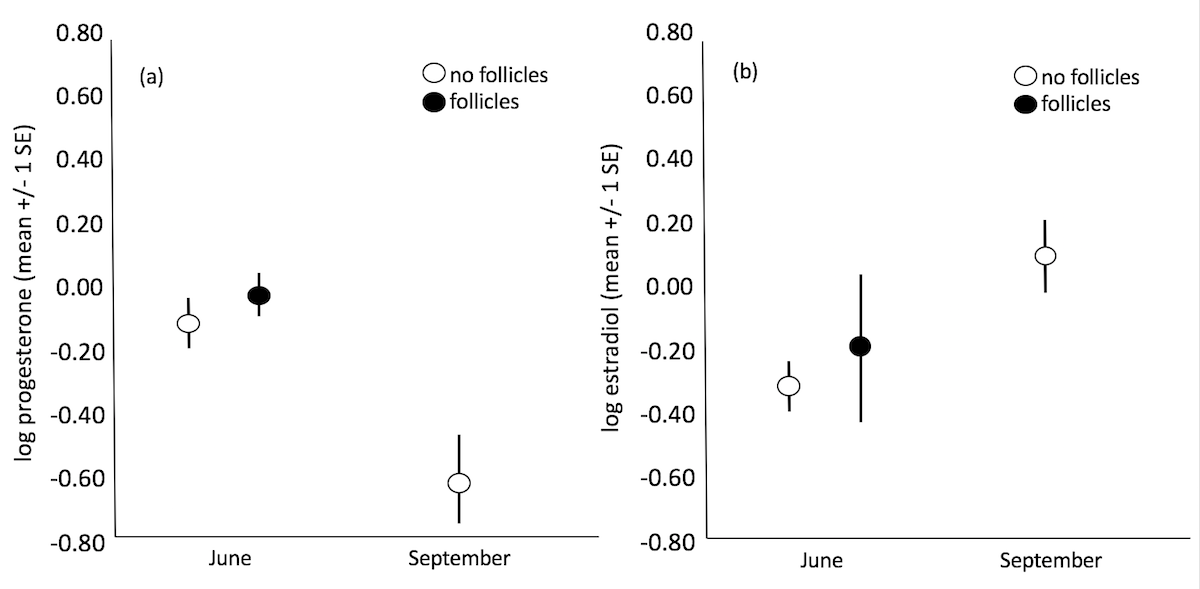

Supplement: suppl_data_coac001 [file suppl_data_coac001.zip › Iguana_SupFig3_UPDATE.tiff]
